# Hair collection protocol in 12-month-old infants

**DOI:** 10.1016/j.cpnec.2024.100243

**Published:** 2024-06-22

**Authors:** Renata Pereira Defelipe, Júlia Terra, Isabella Francischelli, Beatriz Pacheco, Patrícia Pereira Araújo, Ana Raquel Mesquita, Miriam Oliveira Ribeiro, Murilo Correa, Ana Osório

**Affiliations:** a Human Developmental Sciences Graduate Program, Center for Biological and Health Sciences, Mackenzie Presbyterian University, Rua da Consolacao, 930, Building 28, 9th floor, Sao Paulo, 01302-000, Brazil; b ProChild CoLab Against Poverty and Social Exclusion, Campus de Couros, Rua de Vila Flor, n. 166, 4810-225, Guimarães, Portugal; c Human Developmental Sciences Graduate Program, Mackenzie Clinical Analysis Laboratory, Rua da Consolacao, 930, Building 28, 11th floor, Sao Paulo, 01302-000, Brazil

**Keywords:** Hair cortisol, Hair sample collection, Protocol, Infants

## Abstract

**Purpose:**

Most studies assessing hair cortisol were conducted with adults. As specific guidelines for infant hair collection are lacking, we developed a hair collection protocol for 12-month-old infants and assessed its acceptability and feasibility.

**Results:**

Out of the total (*N* = 45), 95.6 % (*n* = 43) of caregivers consented to the procedure, while one caregiver did not consent (2.2 %), and another requested the procedure to be halted before required amount of hair had been reached (2.2 %). Furthermore, two (4.4 %) infants did not have enough hair for collection. There was no attrition due to infant fussiness/crying.

**Discussion:**

We learned five lessons which can help to enhance reproducibility, mother's consent, and mother-infant comfort and acceptance of the procedure. The first lesson is to have the infant sit on the caregiver's lap to ensure the infant feels safe and remains relatively still. The second is to reassure caregivers by showing hair samples representing the amount to be cut as well as by clarifying no unaesthetic gaps would be visible. The third is to caress the infant's head to habituate them to the hair manipulation and to make soap bubbles as distractors. The fourth is to take extra care when securing the lock of hair for cutting because the infant scalp is thin and malleable. The fifth is to place a precision scale in the collection room to ensure the necessary weight is reached.

**Conclusion:**

Our hair collection protocol developed for 12-month-old infants was deemed feasible and acceptable, filled an important literature gap concerning the absence of published protocols for infants, and will contribute to increase the replicability and collection efficiency for other research teams.

## Highlights

• We aimed to fill a literature gap by developing a feasible and acceptable hair collection protocol for 12-month-old infants.
• Out of the total (*N* = 45), 95.6 % (*n* = 43) of caregivers consented to the procedure.
• Lessons for a feasible collection: infant on caregiver's lap; showing hair samples to caregivers; caressing and making soap bubbles; care when pulling the infant hair for cutting.
• Our hair collection protocol will contribute to increase the replicability for other research teams.

## 1 Introduction

Chronic stress and adversity experienced early in life are correlated to a hypothalamus–pituitary–adrenal (HPA) axis hyperactivation which, in turn, is associated with higher risk of physical and mental health problems later in life [1]. Therefore, physiological stress responses, such as provided by cortisol, may constitute a relevant variable to be addressed in developmental studies. Cortisol is a steroid hormone secreted by the adrenal cortex which plays an important role in stress responses throughout the life cycle. Cortisol levels can be assessed in blood (plasma), urine, saliva, and hair. Blood, urine, and saliva samples enable the determination of acute levels, while hair cortisol concentrations (HCC) provide a measure of chronic stress accumulated over the individual's development [2]. According to Wennig [3] hair grows at the approximate rate of 1 cm/month, meaning that each centimeter collected provides information on cortisol secretion from the previous month of an individual's life. However, LeBeau et al. [4] demonstrated a degree of between-subject variability in human hair growth rates (range 0.65–2.2 cm/month), indicating that caution must be taken when interpreting the timing of retrospective cortisol concentrations.

The HCC method has advantages such as relatively non-invasive sample collection and easy storage (room temperature, refrigerators, or freezers) since hair does not degrade easily like other body fluids/tissues [5]. However, two literature reviews have concluded that there are no standard protocols for HCC collection, extraction, and evaluation for studies with humans, and most of the studies: a) were conducted with adults; b) were carried out in high-income countries; c) collected hair from the posterior vertex of the head (cutting as close to the scalp as possible); d) analyzed 3 cm of hair (from 1 to 6 cm); e) used isopropanol for hair washing; f) pulverized the hair strands before extraction; g) performed CLIA, ELISA or LC-MS/MS as the main methods of cortisol analysis [[6], [7], [8]]. A search for relevant studies in the past 10 years on HCC in infants aged around 12 months old and using ELISA as the assay procedure yielded 11 studies (see Table 1).

**Table 1:** Empirical studies measuring hair cortisol concentration (HCC) in infants aged around 12 months (using ELISA).

First author, publication year	N	Country	Infant age (months)	Hair length	Hair amount
Karlén et al. (2015)	209	Sweden	12	3 cm	>3 mg
Liu et al. (2016)	31	USA	9 and 12	3 cm	≥10 mg
Flom et al. (2017)	111	USA	12	3 cm	15–30 mg
Liu et al. (2017)	69	Brazil	12	3 cm	∼50 mg
Hinnouho et al. (2018)	512	Laos	6–23	2 cm	10–20 mg
Bhopal et al. (2019)	712	India	12	2–3 cm	≥10 mg
Ertekin et al. (2021)	49	Turkey	6–15	3 cm	15–30 mg
Tuladhar et al. (2021)	86	USA	12	3 cm	15–30 mg
Winebrake et al. (2022)	90	USA	12	3 cm	15–30 mg
Khalil et al. (2022)	31	USA	6–24	1–2 cm	≥2.5 mg
Tisborn et al. (2023)	72	Germany	15	3 cm	not informed

While HCC has been successfully used as a measure of chronic stress in adults and children, studies with infants pose additional difficulties. Firstly, measuring cortisol in low mass hair samples collected from infants is challenging [20]. Nevertheless, previous literature suggests that cortisol can be accurately measured from hair samples weighing 10 mg [21] and even less, following specific sample manipulations [20]. Another important issue regarding behavioral compliance during hair collection is the fact that infants often resist or refuse having their hair cut due to fear, difficulty in following instructions, and staying still during the procedure. Therefore, important adaptations in standard protocols are needed to ensure that the necessary amount of hair is successfully collected.

However, as most of the published studies investigating hair cortisol in infants are empirical, they do not aim to describe their hair collection protocol in detail. Nonetheless, this is of utmost importance to ensure participant adherence to the procedure, safeguard the well-being of infants, and facilitate the generalization of results. To fill this gap, and given the challenges of hair collection in infants, we provide a step-by-step description of the hair collection protocol designed by our lab for 12-month-old infants, as well as information on acceptability and feasibility of this technique.

## 2 Methods

We developed this hair collection protocol specifically for the 12-month age assessment, as it constitutes a time-point evaluation within a longitudinal study focusing on infant social-emotional development. This study was approved by the Institutional Review Board (CAAE no. 02631818.1.0000.0084). We incorporated guidelines from previous cortisol studies focused mostly on adult samples [8] to define the ideal head collection location, the required length and weight of hair (mg), as well as appropriate labeling and storing conditions [22,23]. We also based our protocol on video-based protocols that demonstrated hair collection procedures in older children and adults [24]. However, existing published protocols did not provide detailed guidance on hair collection skills with infant samples (e.g., where, and how to cut the strands to achieve the minimum weight required for cortisol analysis) or how to manage infant behavior (e.g., fussiness, fear) to increase compliance during the procedure. The few studies conducted with infants around 12 months (Table 2) mostly included a hair length of 3 cm and a hair weight range of 15–30 mg. For this protocol, we followed most studies regarding head collection location (the posterior vertex of the head) and hair length (3 cm). However, concerning the hair weight and storing conditions we decided to be more conservative. Regarding the former, we defined a minimum weight of 30 mg (35–36 mg of hair per infant) because during pilots we had around 18 % of hair loss after pulverization. Regarding storage, considering that most studies are carried out in countries with lower average temperatures and humidity than Brazil, hair samples were kept in the freezer (−20 °C) while awaiting analysis. Below, we detail our guidelines for infant hair collection.

**Table 2:** Hair cortisol concentration (HCC) mean, standard deviation, and range values (pg/mg).

First author, publication year	Infant age (months)	HCC mean (SD)	HCC range (min-max)
Karlén et al. (2015)	12	2.6 (not informed)	0.01–4000.00
Liu et al. (2016)	9 and 12	4.3 (1.3)	not informed
Flom et al. (2017)	12	86.26 (183.63)	3.60–1530.80
Liu et al. (2017)	12	not informed	not informed
Hinnouho et al. (2019)	6–23	28.8 (21.7)	13.80–35.10
Bhopal et al. (2019)	12	not informed	not informed
Ertekin et al. (2021)	6–15	15.11 (11.53)	2.40–46.33
Tuladhar et al. (2021)	12	301.16 (1111.25)	not informed
Winebrake et al. (2022)	12	not informed	not informed
Khalil et al. (2022)	6–24	not informed	3.90–506.40
Tisborn et al. (2023)	15	not informed	not informed

* [[9], [10], [11], [12], [13], [14], [15], [16], [17], [18], [19]].

### 2.1 Pre-collection instructions and setting

The longitudinal study, of which this protocol is a part, comprises three assessments: at 6, 10 and 12 months of infant age. Mothers were informed about the 12-month hair collection procedure (along with other procedures scheduled for the three time points) when presented with the study and invited to sign the informed consent form. Two weeks before the 12-month visit, they received a reminder by text message advising them, if possible, to refrain from cutting the infant's hair to ensure adequate hair length for the analyses. At the lab, mothers received instructions about the hair cortisol procedure and the researchers answered any doubts they might have. When mothers were unsure about the required amount of infant hair and/or concerned about potential unaesthetic gaps, we took the following steps: a) showed samples representing the necessary amount of hair (approximately half the thickness of a matchstick); and b) reminded them that their participation was voluntary, and they could withdraw from the procedure at any time.

**Fig. 1:**
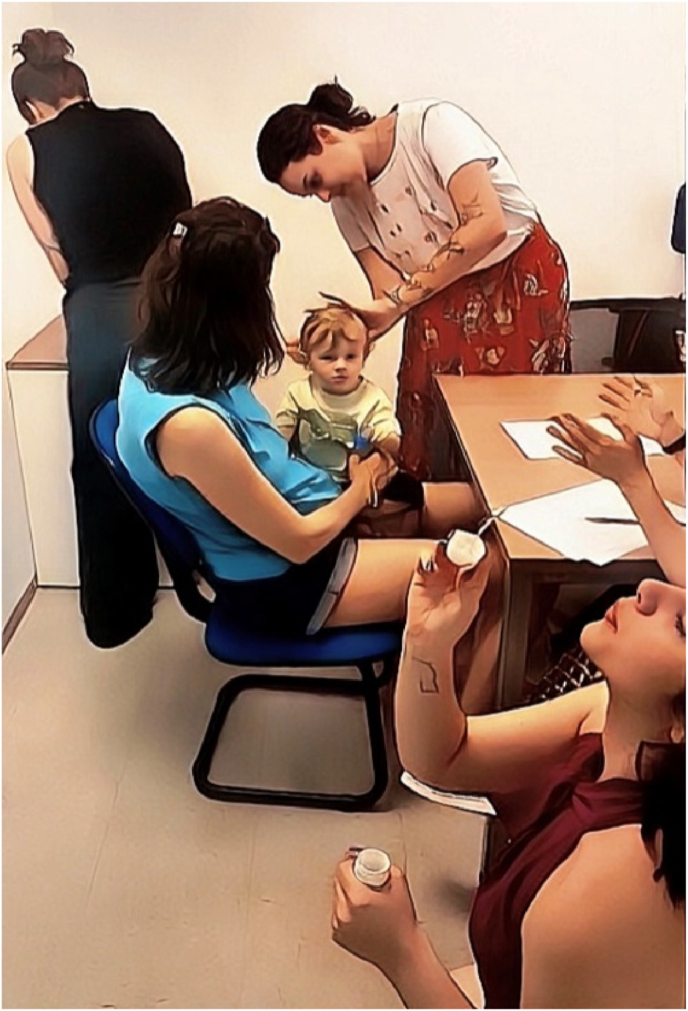
Experimental setting composed of the mother-infant dyad and the experimenters responsible for hair collection (photo published with written consent from the parents).

The procedure took place in a private room at our lab (Fig. 1). The staff was composed of three experimenters: a) Experimenter 1 (E1): responsible for cutting the infant's hair; b) Experimenter 2 (E2): responsible for assisting E1 - holding the hair lock, handing the scissors and other materials, and caressing the infant's head as a distractor; and c) Experimenter 3 (E3): responsible for making soap bubbles during the procedure to distract the infant. All experimenters were previously trained in the procedure and had also experience in interacting with infants/young children.

### 2.2 Materials

**Fig. 2:**
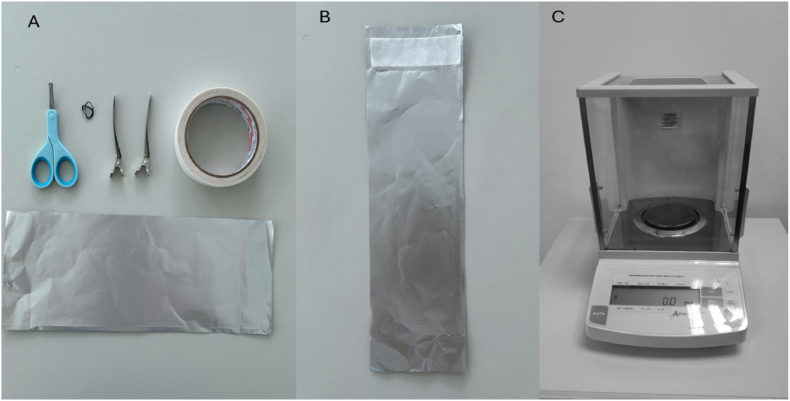
(A, B and C) *A: materials for hair collection. B: aluminum foil with a piece of double-sided tape on one extremity. C: Precision scale.*

Before the hair collection took place, the experimenters' hands and nails were thoroughly cleaned with warm water and soap. The following materials (Fig. 2A) were placed on a tray before the arrival of the participants: blunt scissors with a round tip design (e.g., type used to cut baby nails), double-sided tape, 12 × 30 cm piece of aluminum foil, hair rubber band and hair clips (the former two items if needed, for longer hair). The scissors were disinfected with rubbing alcohol and left to dry before and after each hair collection. Before the procedure, the aluminum foil was prepared with a piece of double-sided tape on one extremity (Fig. 2B). The foil was then placed in a precision scale so that its weight could be tared. Since hair strand density varies greatly especially in children younger than 4 years [8], a precision scale (Fig. 2C) was placed in the room where the hair collection took place, allowing the experimenters to ensure the necessary weight (in mg) had been reached before the dyad left the lab.

### 2.3 Hair collection protocol in 12-month-old infants

The child sat comfortably on their mother's lap on a 90-degree angle, allowing for their head to be manipulated by the experimenters (Fig. 1). E3 would then begin distracting the child using soap bubbles. If necessary, the mother's assistance (to distract or talk to the child) would also be requested. Once the infant was settled in the mother's lap and paying attention to E3, the hair collection procedure would begin, following these steps.

1. E1 and E2 would position behind the infant and begin gently touching/caressing their head and hair, so that they would get used to being touched in the head. If the infant turned around, the experimenters would smile and say “Hello!“, then gently redirect the child's attention towards E3 (e.g., “Look at that big bubble! Did you see that?“) (Fig. 3B).

**Fig. 3:**
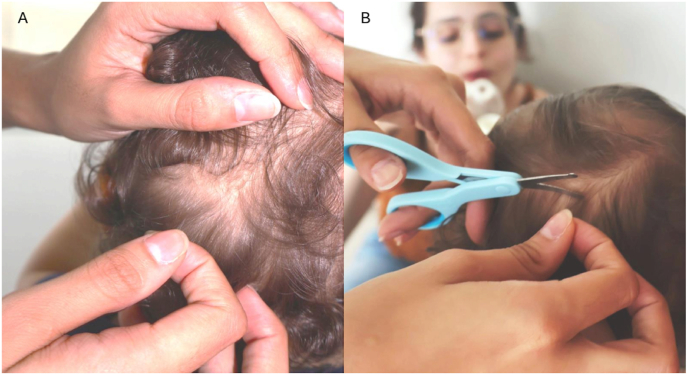
(A and B) A: E2 manually parts hair sections.B: E1 cuts the lock of hair as close as possible to the scalp (posterior vertex of the head), while E3 is in the background making soap bubbles as a distractor.
2. E2 proceeded to manually part hair sections from the back of the infant's head until the roots were visible (Fig. 3A).
3. E2 selected a small lock of hair (Fig. 3A), secured it with their fingertips pulling it very slightly, and then E1 proceeded to cut the hair as close as possible to the scalp, always from the posterior vertex of the head (i.e., the line below the ear) (Fig. 3B). Extra caution needs to be taken when securing the lock for cutting, because if the hair is pulled too strongly, it may cause discomfort to the child. Importantly, given that the infant scalp is thinner and more malleable than the adult scalp, it may be further extended when pulling the hair, so it is important to always check if there is no skin in the path of the scissors when cutting, to avoid any scalp laceration.
4. Different from hair collection in adults, where a hair lock is collected from only one specific spot of the head, considering the low mass of infant hair, cuts were done in different places of the infant's head (along an invisible line across the posterior vertex, i.e., from ear to ear), all close to the scalp.

**Fig. 4:**
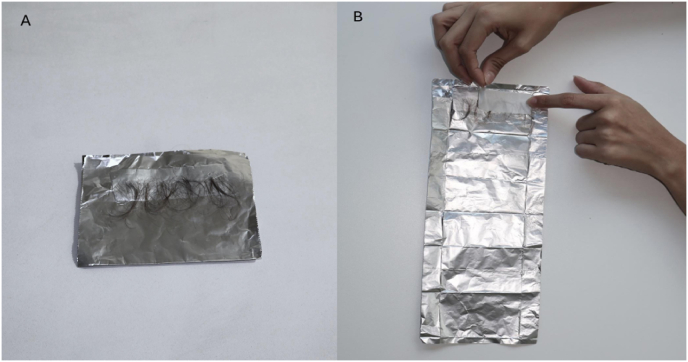
(A and B) Locks of hair placed with the root side up onto the double-faced tape in the aluminum foil.

5. The cut hair was placed with stainless steel tweezers in a previously tared aluminum foil inside the precision scale (Fig. 2C) to confirm the necessary amount of hair (35–36 mg).
6. Locks of hair were placed with the root side up onto the double-faced tape in the aluminum foil using stainless steel tweezers (Fig. 4A). This would ensure that the researchers knew which section was closer to the scalp when later cutting the 3 cm segments. The plastic covering the double-sided tape was then glued to the tape segment, over the hair, to avoid difficulty when later opening the packet (Fig. 4B).

### 2.4 Sample storage considerations

**Fig. 5:**
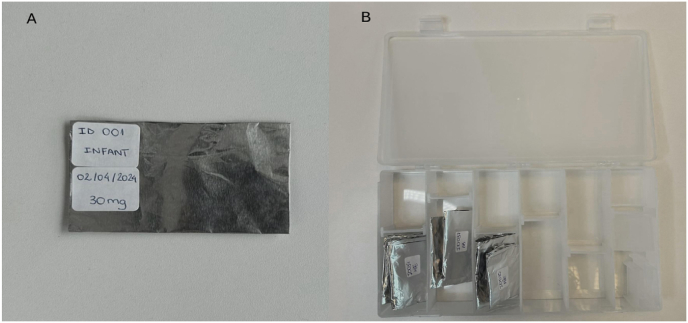
(A and B) A: Identification of hair locks. B: Storage of hair locks.

Once the necessary amount of hair had been obtained, the researchers would fold the aluminum foil in an envelope-like manner, keeping the hair secure inside. A tag containing the unique code attributed to that infant as well as the collection date (e.g., ID 001 April 02, 2024) would then be sticked to the envelope (Fig. 5A). After that, all identified samples were stored in a box (Fig. 5B) inside a freezer at −20 °C.

### 2.5 Hair cortisol concentration (HCC)

The literature underlines the need of establishing HCC reference ranges across all age groups, with particular emphasis on children under the age of 4 years. These age groups merit additional attention due to the significant and varied changes in their hair characteristics [8]. There is substantial variation in the HCC values reported, with HCC values ranging (when informed) from 0.01 to 4000 pg/mg [9]. Mean values (when informed) ranged from 2.6 [9] to 301.16 pg/mg [16] (Table 2).

### 2.6 Analysis for acceptability and feasibility

Acceptability of our infant hair collection protocol was assessed based on the caregiver's consent to the hair collection procedure as well as infant's behaviors conducive to successful data collection. Feasibility was evaluated based on successful collection of the necessary amount of hair.

## 3 Results

We collected hair for cortisol assay from September 2023 to May 2024. Out of a total of 45 12-month-old infants, one caregiver (2.2 %) declined to consent to the procedure and another caregiver (2.2 %) requested that the procedure be halted before the required amount of hair had been reached. Furthermore, two (4.4 %) infants did not have enough hair for collection. No collection was unsuccessful due to infant fussiness or continued crying. All caregivers were the infants’ mothers.

## 4 Discussion

We developed a hair collection protocol specifically for 12-month-old infants. We aimed to provide a step-by-step description of a protocol for infants considering two main points. First, there are substantially less studies assessing hair cortisol in infants and children, compared with adults [9]. Second, most of the published studies investigating hair cortisol in infants are empirica, not aiming to thoroughly describe the hair collection protocol.

We learned the following lessons during the development of this protocol.

1. We found that asking the mother to put the infant on her lap was a good strategy to ensure a sense of security, avoid fear, and increase the likelihood of the infant staying still during the hair cutting procedure.
2. When mothers expressed uncertainty and concern regarding potential aesthetic gaps resulting from the procedure, presenting samples of hair locks to be cut (approximately half the thickness of a matchstick) and ensuring that hair was trimmed from various locations on the infant's posterior vertex (always close to the scalp) helped alleviate their concerns and encouraged them to consent to the procedure.
3. Caressing the infant head before and throughout hair cutting and making soap bubbles during all the procedure were effective as distractors and lead to increased infant compliance during the procedure.
4. Extra caution needed to be taken when securing the lock of hair for cutting, because the infant scalp is thinner and more malleable than the adult scalp. Pulling the hair could inadvertently strain the scalp, causing discomfort to the infant or even leading to risk of scalp laceration.
5. Considering the low mass of infant hair, we placed a precision scale in the hair collection room to monitor the required weight (in mg) immediately after collection and before the dyad left the lab.

## 5 Conclusion

Given the high rates of both maternal consent and infant compliance to the procedure, our hair collection protocol for 12-month-old infants was deemed acceptable and feasible. Furthermore, it filled an important literature gap concerning the absence of published protocols on hair collection in infants. We suggest that empirical studies incorporate more detailed information on their hair collection and cortisol extraction protocols for infants to ensure that procedures and methods are comparable and standardized. For instance, most of the studies have been conducted in countries with lower average temperatures and humidity than Brazil (e.g., Sweden [9]; Germany [19]). As far as we know, the variation in humidity and temperature parameters is not controlled in these studies. Thus, we took the precaution of storing the hair samples in aluminum foil (not in plastic microtubes) in the freezer (−20 °C). Further studies may explore whether storing conditions may need to vary according to regional climatic conditions. Moreover, Noppe et al. [8] claimed there is an important gap in the literature concerning HCC reference ranges measured in all ages, especially in children under the age of 4 years. In order to address this gap, future studies are needed to investigate how cortisol deposition takes place and why there is such a variability in children under the age of 4, also providing a step-by-step guideline for hair sample collection. This would: a) increase the replicability and collection efficiency for the research teams; b) decrease uncertainty and promote participant compliance; and c) provide practical strategies for ensuring the safety and comfort of mother-infant dyads during the procedure.

## Role of the funding source

This study was supported by three funding agencies: 1) São Paulo Research Foundation (FAPESP - Brazil) (no. 2021/06693-4: Ana Osório; no. 2023/04029-5: Renata Defelipe; no. 2023/10038-7: Júlia Terra; no. 2023/09990-5: Isabella Francischelli; no. 2023/16192-8: Beatriz Pacheco); 2) Mission Interface Program from the Resilience and Recuperation Plan, approved by ANI - Agência Nacional de Inovação, S.A. (Portugal) (no. 01/C05-i02 /2022); and 3) CAPES/Proex (no. 0426/2021) and CAPES/PrInt (no. 88887.310343/2018-00) (Brazil).

## CRediT authorship contribution statement

**Renata Pereira Defelipe:** Writing – review & editing, Writing – original draft, Visualization, Validation, Supervision, Methodology, Formal analysis, Conceptualization. **Júlia Terra:** Writing – review & editing, Writing – original draft, Visualization, Validation, Methodology, Investigation, Formal analysis, Conceptualization. **Isabella Francischelli:** Writing – review & editing, Visualization, Validation, Methodology, Investigation. **Beatriz Pacheco:** Writing – review & editing, Visualization, Validation, Methodology, Investigation, Formal analysis. **Patrícia Pereira Araújo:** Writing – review & editing, Validation, Resources, Investigation. **Ana Raquel Mesquita:** Writing – review & editing, Supervision, Funding acquisition. **Miriam Oliveira Ribeiro:** Writing – review & editing, Supervision, Resources. **Murilo Correa:** Writing – review & editing, Resources. **Ana Osório:** Writing – review & editing, Writing – original draft, Visualization, Validation, Supervision, Project administration, Methodology, Funding acquisition, Conceptualization.

## Declaration of competing interest

The authors declare no competing interests.
